# Acceptability and effectiveness of a study information video in improving the research consent process for youth: a non-inferiority trial

**DOI:** 10.1136/bmjgh-2023-014481

**Published:** 2025-01-11

**Authors:** Tinashe Cynthia Mwaturura, Victoria Simms, Ethel Dauya, Som Kumar Shrestha, Salmaan Ferrand, Talent Shavani, Chido Dziva Chikwari, Constance R S Mackworth-Young, Tsitsi Bandason, Constancia Mavodza, Mandikudza Tembo, Katharina Kranzer, Sarah Bernays, Rashida Abbas Ferrand

**Affiliations:** 1 The Health Research Unit Zimbabwe, Biomedical Research and Training Institute, Harare, Zimbabwe; 2 MRC International Statistics & Epidemiology Group, London School of Hygiene & Tropical Medicine, Department of Infectious Disease Epidemiology, London, UK; 3 Human Development Report Office, United Nations Development Programme, New York, New York, USA; 4 Department of Global Health and Development, London School of Hygiene & Tropical Medicine, London, UK; 5 Clinical Research Department, London School of Hygiene & Tropical Medicine, London, UK; 6 School of Public Health, University of Sydney SDN, Sydney, New South Wales, Australia

**Keywords:** Health policy, Intervention study, Public Health

## Abstract

**Introduction:**

Obtaining informed consent for research includes the use of information sheets, which are often long and may be difficult for participants to understand. We conducted a trial to investigate whether consent procedures using a study information video coupled with electronic consent were non-inferior to standard consent procedures using participant information sheets (PIS) among youth aged 18–24 years in Zimbabwe.

**Methods:**

The trial was nested within an endline population-based survey for a cluster-randomised trial from October 2021 to June 2022. Randomisation of participants to video or paper-based consent was at household level. We assessed non-inferiority in comprehension of the study using a questionnaire. The video method was accepted as non-inferior to standard consent procedures if the 95% CIs of the mean difference did not fall below the prespecified margin of 1.98. Thematic analysis was conducted on brief qualitative discussions with randomly selected youth to explore the acceptability of video and PIS within consent methods.

**Results:**

Overall, 921 participants were enrolled (54% female). The median age was 20 (IQR 18–24) years. The mean comprehension score was 25.4/30 in both arms. The mean difference in comprehension between arms was −0.02 (95% CI −0.51 to 0.47) showing non-inferiority of the intervention in comprehension of study information. Youth (N=90) described both consent methods as interactive and inclusive. Those in the video consent arm felt it was exciting and youth focused. The use of imagery to explain procedures strengthened the perceived trustworthiness of the research. However, the high volume of information in both arms reduced acceptability.

**Conclusion:**

Comprehension of study information using an information video is non-inferior to a paper-based consent method. Using information videos for consent processes shows promise as a person-centred and context-sensitive approach to enhance the informed consent process and should be encouraged by ethics committees.

**Key messages**

**What is already known on this topic**

- Informed consent is core to ethical conduct of research, which requires true understanding of research by participants. Procedural requirements of ethical review boards for consent forms often result in lengthy information sheets and use of jargon, which may hinder meaningful comprehension of research information.

**What this study adds**

- We investigated whether comprehension of information provided by video was non-inferior to the traditional paper-based participant information sheets. In this study, comprehension of research information provided by video was as good as that from paper information sheets. The video promoted engagement with research information and trust in the research process.

**How this study might affect research, practice or policy**

- The video consent method has the potential to enhance the informed consent process to becoming person-centred and context-sensitive and should be considered as a valuable option by ethical review boards.

## Introduction

 Conducting inclusive and ethical research is crucial to improving public health.^1^ Core to the ethical integrity of research is ensuring that an individual’s decision about whether to participate in a research study is based on a clear understanding of the purpose of the research, its activities and the potential implications of their participation. This is enshrined within the principles of the informed consent process, which require that potential participants be given appropriate information about the research in a comprehensible manner without coercion or inappropriate inducement.^2^ These are universally important within research but may be particularly vital where structural conditions exacerbate populations’ vulnerability to research exploitation due to poverty, illiteracy, social exclusion or limited access to health services.^3 4^

While considerable efforts are made to prevent coercion of potential participants, there has been less interrogation into whether the information provided in the consent process is genuinely comprehensible and is in an accessible format to individuals being recruited.^5^,^8^ The procedural requirements of ethical review boards for consent forms commonly result in lengthy participant information sheets (PIS) and use of lexicon, which may hinder comprehension of the proposed research.^6 9 10^ Concerns have been raised that the documents are produced to satisfy the demands of the institutional review boards, without due consideration of how to balance the needs of participants to be given information in formats that would enable their adequate understanding and informed decision-making.^6 11 12^ This can be partially redressed if the researcher reads the information sheet with potential participants and engages in a dialogue about the study, but this method relies on consistent application and is difficult to monitor.^13^,^16^

In response, there have been growing calls to be more innovative in communication within the consenting process.^17 18^ One approach is the use of video-based delivery. Research exploring the use of videos to support informed consent in Uganda, South Africa^19 20^ and The Gambia^21^ indicates that it may improve information recall and may be acceptable to a range of stakeholders.^17^ Presenting information about a study in a visual format may potentially circumvent challenges in explaining complex procedures by demonstrating them, making them more comprehensible and engendering trust in the research. This may ultimately improve engagement with and acceptability of research.

We aimed to investigate whether a consent procedure that used a video to deliver all the information about a study including research procedures was non-inferior to standard consent procedures using a paper-based PIS. We compared the comprehension of study information (the primary outcome), time taken to complete consent process and the acceptability of consent method.

## Methods

### Study design and setting

A non-inferiority trial with a qualitative evaluation of the consent procedures was embedded within a population-based survey of youth aged 18–24 years in Zimbabwe. The survey was undertaken to ascertain the outcome of a cluster-randomised trial (CHIEDZA) that investigated community-based integrated HIV and sexual and reproductive health services for youth (*CHIEDZA Trial registration number NCT 03719521*).^22^

The survey was conducted in three provinces of Zimbabwe (Harare, Bulawayo and Mashonaland East (M. East)) between October 2021 and June 2022 with eight clusters (defined as geographically demarcated areas) per province. An anticipated sample of 700 youth were to be recruited per cluster (total 16 800 participants). The survey start in each province was staggered by 3 months with the clusters in Harare surveyed between October and December 2021, Bulawayo clusters from January to March 2022 and M. East clusters from April to June 2022. Clusters were mapped and randomly selected sections of street were enumerated, with every individual in the enumerated households aged 18–24 years and able to provide consent eligible to participate in the survey. The survey involved an interviewer-administered questionnaire that collected sociodemographic data, and data on sexual behaviour, HIV testing and treatment history, experience of violence, alcohol and substance use, mental health, vaccination against SARS-CoV2 and access to digital technology. Height, weight and three blood pressure measurements were taken, a dried blood spot was collected for measurement of HIV antibodies and HIV viral load. In Harare and Bulawayo, a urine sample for testing for sexually transmitted infections (STI) was collected from a subset of participants on randomly selected days to ascertain the outcomes of a nested trial of STI screening.^23^

### Consent trial procedures

All individuals eligible for the survey were also eligible to participate in the non-inferiority trial (online supplemental figure 1). Participants could take part in the consent trial but opt out of participating in the survey. A subset of households were randomly selected to participate in the trial. The trial was conducted within the second month of the implementation of the survey in each province. A randomisation list to randomise households to either the control or intervention arm was generated using STATA (V.17.0 software) and was uploaded onto a tablet such that when a household identification number was allocated the study arm would be assigned to the household. All eligible individuals in a household were allocated to the same arm. Individuals were asked whether they would like to participate in the non-inferiority trial and gave verbal consent. The researcher went through the process of giving research information using either the PIS and the video while all the eligible household members were present. However, consent to participate in the trial was obtained separately. Likewise participant comprehension of research information was assessed individually.

Individuals in the control arm of the trial were given information about the research using either a two-page PIS in English or three-page PIS in Shona or Ndebele designed using the standard template of the Medical Research Council of Zimbabwe (https://www.mrcz.org.zw/guidelines/). The researcher carried out the consent process in the preferred language of the participant. The standard procedure was for the researcher to read the PIS, and in a few instances, participants opted to read for themselves. Participants consented to participation using a standard two-page consent form, each attached to the PIS. One copy of the consent form and PIS was retained by the study staff member and the other by the participant.

### Consent trial intervention

Intervention arm participants were shown a video on a tablet containing all the information on the PIS. The film enacted the study procedures illustrated by the study staff, most of whom were aged between 18 and 24 years. The videos visualised the information given in the standard information sheet and included animations to aid comprehension. The initial script was drafted in English and translated into Shona and Ndebele and back-translated into English. The videos were piloted with youth from study communities and youth researchers, and the content and the terminology were iteratively refined taking account of their feedback. The animations were developed by the study team. The video was developed by a professional videographer and editor, who worked collaboratively with the study team. The cost of development of the videos was approximately US$500. The video was available in English (7 min 5 s and 8 min 11 s), Shona (9 min 55 s and 8 min 29 s) and Ndebele (11 min 54 s and 10 min 12 s), each with two versions, respectively. The second version of the video included information on an additional survey procedure of urine sample collection to test for STIs for a subset of participants.

While the informational content of the video was consistent, the duration of the video differed due to the variation in the specific linguistic characteristics across the three languages, with Shona and Ndebele using more words to explain the same concepts as in English. Participants could rewind the video or pause at any stage to ask questions. Participants were also given a sheet containing the Uniform Resource Locator (https://www.chiedza.co.zw/endlinesurvey) of the videos and the contact details of the study team. Participants consented to participation on an electronic tablet (maintained by the research team), and a paper copy of the consent form was given to participants for their records.

Participants were given the opportunity to ask questions during and after the process of being given information in both arms.

### Patient and public involvement statement

In the CHIEDZA trial, we interacted with young people aged 16–24 years for 30 months from which we learnt that young people do not enjoy reading research information sheets. This motivated us to develop better ways to communicate research information with young people. Participants were not involved in the design of this study and the recruitment and conduct of the study. In the developmental phase of the consent videos, 10 young people who participated in the CHIEDZA trial watched the videos to assess the acceptability of the consent videos including the time taken to go through the videos, what they liked and did not like on the videos. The study findings will be added to the CHIEDZA website, (https://www.chiedza.co.zw/endlinesurvey). Young people will be alerted to view the findings on the CHIEDZA website through social media platforms such as X and Facebook.

### Ascertainment of trial outcomes

The primary outcome of the trial was the mean comprehension score ascertained through a 30-item questionnaire administered immediately after the delivery of information about survey procedures. The questionnaire was designed in English; however, the researchers were also trained to administer the questionnaire in the two local languages. Responses were recorded on an Android tablet using SurveyCTO Collect (Dobility, Washington, USA). The structure and format of the comprehension questionnaire were adopted from previous studies, which sought to investigate informed consent comprehension of study information in South Africa^19^ and The Gambia.^13 21 24^ In these studies, the context of the comprehension questionnaires was specific to the study information, therefore, we developed the comprehension tool with each question designed to assess comprehension of the outcome survey information. The information assessed included why individuals were invited to participate, what study procedures were to be undertaken, if participation was voluntary, who had access to their health information and if money was given for taking part in the study. The 30 items included 26 close-ended questions (responses yes, no and I don’t know), three questions with one correct answer and three incorrect answers and one question with multiple correct answers and three incorrect answers. The correct responses to the close-ended and multiple-choice comprehension questions were scored 1 and the wrong and/or ‘I don’t know’ responses were scored 0. The comprehension score is a continuous outcome with a maximum score of 30 for all correct answers and a minimum of zero. Six of the 30 items were related to consent principles, and participant scores on these six items were calculated as a subscale.

The secondary outcome was the time taken to complete the consent process. The process start and end times were automatically captured on a tablet. The start time was marked as the time when the researcher opened the consent form on Survey CTO Collect to determine the household’s assigned arm and ended when the participant indicated whether they would participate in the survey or not.

### Statistical considerations

Analyses were conducted using STATA V.17.0 software (Statcorp, Texas). The comprehension score and time taken to consent were calculated as continuous variables and were summarised as means (SD). Categorical variables were summarised as counts (percentages). Descriptive characteristics and the total comprehension scores of the participants were distributed overall and by trial arm. We assessed for non-inferiority of the video method compared with the paper method. Non-inferiority trials usually aim to preserve a proportion of the effect of the active control against a placebo. In this case, there is no placebo for consent, hence we estimated the effect size using a systematic review of trials comparing similar intervention groups.^14 25^ We used the pooled estimate for comparing enhanced consent form versus control (usual consent method) for the standard studies that reported pooled estimates of the standard mean difference (SMD) as 1.47 (95% CI 0.7 to 2.23). We used the lower boundary of the 95% CI (SMD=0.7) to set the preliminary margin, and the final margin was set at 75% of the preliminary margin to preserve the minimum effect. The SD of the primary outcome in the dataset was 3.77. Therefore, the final margin was 3.77×0.75×0.7=1.98. We also conducted sensitivity analysis against margins 1.41 and 0.90.

The comprehension scores mean difference is robust to non-parametric distributions of the underlying data. As a secondary outcome, the comprehension scores were reclassified to a binary variable based on the median, with scores above the median classified as ‘above average comprehension’ to reflect participants who scored better than at least 50% of the participants. We estimated the relative risk ratio for above average comprehension for the paper-based information versus video information and the 95% CI using Poisson regression with robust error variance.^26^ We arbitrarily set the non-inferiority margin at RR=1.20 and we conducted sensitivity analysis at a more restricted margin of RR=1.10.

Assuming a two-sided CI at the alpha of 0.05, a sample size of 460 in each arm, and an SD of 3.77, the study had nearly 100% power to detect the difference in the primary outcome of 1.98 units and 1.41 units, and about 90% power to detect the difference of 0.90 units.

Univariate and multivariate logistic regression was used to assess the association of sociodemographic variables (age, sex, province, highest education level and main income generating activity) with comprehension separately in the intervention and control arms, adjusted for clustering by household.

We used the Consolidated Standards of Reporting Trials checklist when writing our report.^27^

### Qualitative methods and analysis

A total of 90 participants in Harare, Bulawayo and M. East were engaged in brief qualitative discussions on the same day of the consenting process to assess the acceptability of the consent method they received. Field researchers who were trained in qualitative methods invited all the participants that they engaged with on the STI testing days to participate in the qualitative discussions. Interviews were conducted until thematic saturation was reached. As the field researchers were visiting households allocated to the study at random, the qualitative sample reflected this random selection. Random selection of STI testing days was performed using random allocation in STATA, stratified by province. Discussions were conducted in the preferred language of the participant. The researchers asked the participant open-ended questions about their thoughts about the consenting process, including the method, what they liked and did not like or understand, their opinion on the relative importance of understanding study information in deciding to take part in the study and who (ie, population groups or types) they thought the consent method that they had engaged in would work well for, or not. The researcher noted down the points raised in the discussion when the participant stepped out to provide the urine sample for STI testing, and summarised the discussion on an audio recorder immediately after leaving the household of the participant. Field researchers were trained in the accurate reporting of the discussions^28^ and discussion summaries had a length of approximately 500 words.

Data collection and analysis were conducted iteratively. The summaries of the qualitative discussions were transcribed for analysis. Thematic analysis was conducted by a qualitative research assistant (TS) who participated in the data collection in the Harare province, supported by senior social scientist (SB). Each transcript was reviewed to obtain the emerging themes from the qualitative discussions. Using Microsoft Excel, the emerging themes were listed with corresponding statements and demographic data for each participant.

### Ethical considerations

Ethical approval for the CHIEDZA outcome survey and the consent trial was obtained from the Medical Research Council of Zimbabwe (MRCZ/A/2387) and the Institutional Review Boards of the Biomedical Research and Training Institute (AP149/2018) and the London School of Hygiene and Tropical Medicine (16 124). The ethics committees waived requirements for written consent and participants provided verbal consent to participate in the consent trial.

### Grant information

The CHIEDZA trial in which the non-inferiority trial was embedded is funded by the Wellcome Trust (Senior Fellowship to RAF: 206316/Z/17/Z). The funders had no role in study design, data collection and analysis, decision to publish or preparation of the manuscript.

## Results

**Figure 1:**
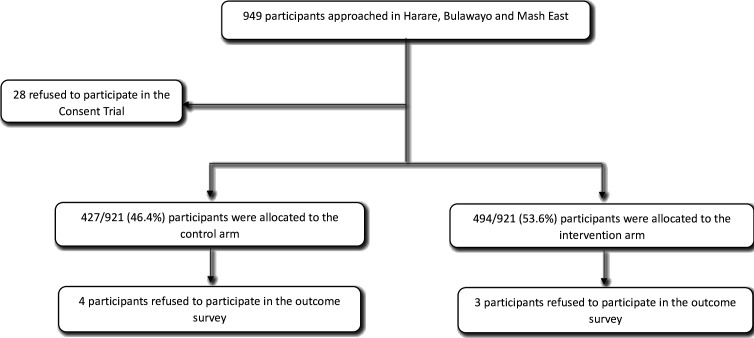
Flowchart of participants in the study.

Of the 949 youth that were approached, 921 (97%) agreed to participate in the consent trial (figure 1). All 28 participants who refused to participate in the trial agreed to participate in the survey. Of the 921 participants who participated in the trial, 4/427 in the control arm and 3/494 in the intervention arm declined participation in the outcome survey. Most (80.7%) of the households had one eligible participant; the maximum number of participants per household was four (0.1%). The median age of participants was 20 (IQR 18–24) years and 54% were female. Participant characteristics were largely balanced between arms, except that a higher proportion of control arm participants were recruited from Bulawayo relative to M. East compared with the intervention arm (table 1).

**Table 1:** Baseline characteristics of study participants by study arm

Characteristic	Intervention (%)(n=494)	Control (%)(n=427)
Age (years)		
18–20	260 (52.6)	222 (52.0)
21–24	234 (47.4)	205 (48.0)
Sex		
Female	271 (54.9)	226 (52.9)
Male	222 (44.9)	200 (46.8)
Intersex	1 (0.20)	1 (0.23)
Province		
Harare	175 (35.4)	152 (35.6)
Bulawayo	144 (29.2)	145 (34.0)
M. East	175 (35.4)	130 (30.4)
Highest education level *		
Primary	20 (4.10)	19 (4.50)
Secondary	446 (90.8)	383 (90.5)
Tertiary	25 (5.10)	21 (5.00)
Main activity *		
None	274 (55.8)	254 (60.1)
Going to school	107 (21.8)	97 (22.9)
Employed	28 (5.70)	22 (5.20)
Informal work	82 (16.7)	50 (11.8)
Previous research study participation		
No	369 (74.7)	309 (72.4)
Yes	125 (25.3)	118 (27.6)

* Missing data for 7seven participants.

### Trial outcomes

The mean comprehension score was 25.4 in the control arm and 25.4 in the intervention arm (SD 3.8). The mean difference in the comprehension score between the control and intervention arms was −0.02 (95% CI −0.51 to 0.47). The upper confidence limit of the mean difference was well below the non-inferiority margin of 1.98, thus the non-inferiority of the intervention to the control is established. Non-inferiority was also shown in sensitivity analysis using the thresholds of 1.4 and 0.9. The relative risk (RR) of comprehension in the control arm compared with the intervention arm was 0.97 (95% CI 0.88 to 1.08). The upper margin of the CI of the RR is well below the inferiority margin of RR=1.20, thus the non-inferiority of the intervention compared with control is exhibited in the trial. In sensitivity analysis, non-inferiority was also shown at the margin of RR=1.10.

**Figure 2:**
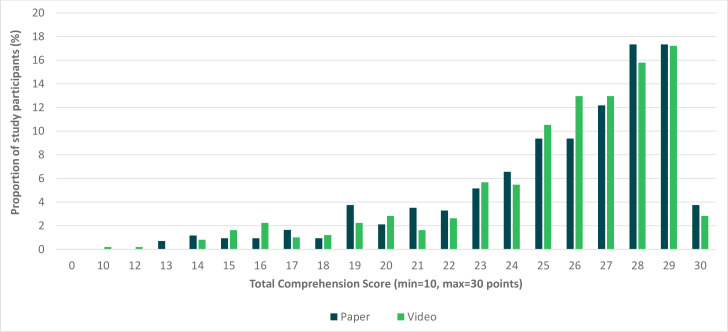
Distribution of total comprehension scores by study arm.

Over 15% of participants in each arm scored ≥28 points and less than 4% of the participants scored ≤22 points in both arms (figure 2). Almost 95% of the participants understood the reason for being invited into the study while 14.7% of the participants did not comprehend the reason for signing the consent form (onlinesupplemental table 1^ 2^).

The median comprehension score was 26 (IQR 24–28). Education level and main activity status (employment or education) were not associated with comprehension in both the intervention and control arm. However, intervention arm participants aged 18–20 years had 40% higher odds of above average comprehension than the 21–24 year olds (adjusted OR (aOR) of 0.60; 95% CI (0.41 to 0.87), p=0.007). Additionally, participants who lived in Harare had 57% and 38% increased odds of above-average comprehension compared with the participants who lived in Bulawayo (aOR 0.43; 95% CI (0.26 to 0.70)) and M. East (aOR of 0.62; 95% CI (0.39 to 0.97), p=0.002), respectively. Among the control arm, comprehension was not associated with age and province. However, participants who participated in a previous research study had two times the odds of above-average comprehension than those who had not participated in a research study before the consent trial (table 2).

**Table 2:** Association of baseline characteristics with mean comprehension scores in control arm and intervention arm participants

	Control arm (N=427)					Intervention arm (N=494)				
Characteristics	% with comprehension score>mean	Univariate analysis		Multivariate analysis*		% with comprehension score>mean	Univariate analysis		Multivariate analysis*	
	N (%)	OR (95% CI)	P	OR (95% CI)	P		OR (95% CI)	P	OR (95% CI)	P
Age (years)										
18–20	131 (59.3)	Ref		Ref		175 (67.3)	Ref		Ref	
21–24	124 (60.5)	1.05 (0.71 to 1.55)	0.800	0.96 (0.64 to 1.43)	0.833	129 (55.4)	0.60 (0.42 to 0.87)	0.006	0.60 (0.41 to 0.87)	0.007
Sex										
Male	112 (56.0)	Ref		Ref		139 (62.6)	Ref		Ref	
Female	143 (63.3)	1.35 (0.91 to 2.00)	0.126	1.26 (0.83 to 1.90)	0.276	165 (60.9)	0.93 (0.65 to 1.34)	0.695	0.81 (0.55 to 1.20)	0.293
Province										
Harare	94 (61.8)	Ref		Ref		123 (70.3)	Ref		Ref	
Bulawayo	78 (53.8)	0.72 (0.45 to 1.14)		0.84 (0.51 to 1.36)		76 (52.8)	0.47 (0.30 to 0.75)		0.43 (0.26 to 0.70)	
M. East	83 (64.3)	1.11 (0.68 to 1.81)	0.171	1.26 (0.76 to 2.07)	0.280	105 (60.3)	0.64 (0.41 to 1.00)	0.005	0.62 (0.39 to 0.97)	0.002
Highest education level †										
Primary	8 (42.1)	0.46 (0.18 to 1.17)		–	–	12 (60.0)	0.92 (0.37 to 2.28)		–	–
Secondary	234 (61.3)	Ref				277 (62.1)	Ref			
Tertiary	12 (57.0)	0.84 (0.35 to 2.05)	0.250			14 (58.3)	0.85 (0.37 to 1.97)	0.920		
Main activity †										
None	150 (59.3)	Ref		–	–	164 (59.9)	Ref		–	–
Going to school	60 (61.9)	1.11 (0.69 to 1.80)				67 (63.2)	1.15 (0.73 to 1.83)			
Employed	10 (45.5)	0.57 (0.24 to 1.37)				20 (71.4)	1.67 (0.71 to 3.94)			
Informal work	34 (68.0)	1.46 (0.77 to 2.78)	0.650			52 (63.4)	1.16 (0.70 to 1.94)	0.620		
Previous study participation										
No	169 (54.9)	Ref		Ref		225 (61.1)	Ref		–	–
Yes	86 (72.9)	2.21 (1.39 to 3.51)	0.001	2.25 (1.40 to 3.60)	0.001	79 (63.2)	1.09 (0.72 to 1.66)	0.682		

* Multivariate analysis adjusted for age, sex and province; ʂ Missing data for 7 participants.

† Missing data for seven participants.

The mean time taken to complete the consent process was 4.30 min (SD 3.10) using the paper and 11.8 min (SD 6.25) using the information video (online supplemental figure 2).

### Acceptability of methods of receiving information about research studies

A total of 90 participants (60 from Harare, 20 from Bulawayo and 10 from M. East) were engaged in the qualitative discussions with 43 in the control arm and 47 in the intervention arm. Thematic saturation was identified within Harare clusters, and further data collection was conducted in Bulawayo and M. East to check the conceptual generalisability of the qualitative findings. There was no difference in the characteristics and mean comprehension score (27.0 points, SD 2.3) by study arm of the participants who took part in the qualitative discussions (online supplemental table 3).

**Figure 3:**
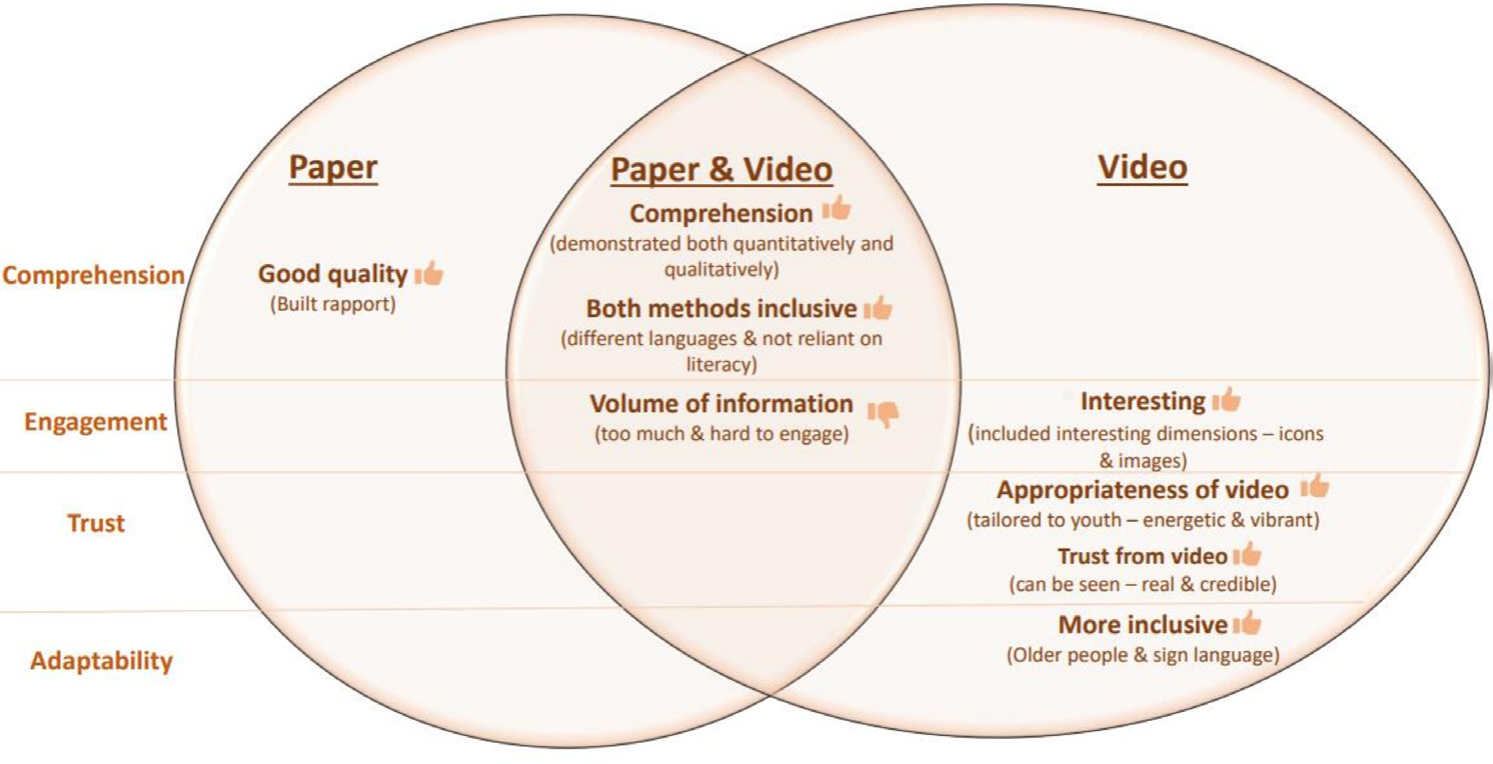
Qualitative findings exploring acceptability.

Participants evaluated the acceptability of the consenting process (in the arm they were assigned to) through four domains: comprehension, engagement, trust and adaptability (figure 3). As participants only experienced one approach, a direct comparison was not feasible. Despite the fact that each approach was considered acceptable, those in the intervention arm (video consent) emphasised features which they highly appreciated which were unique to this approach and indicated a higher degree of acceptability.

#### Comprehension

Participants in both arms qualitatively described similar levels of confidence in how comprehensible they found the information presented. Participants described how they liked being able to choose their preferred language from a range of options, which supported their ability to understand the content.

I liked that the video was played in Shona which is the language I understand better because if it was played in English I was not going to fully understand owing to my challenges understanding English (C14211—video; male; 18 years).

I liked that you asked me which language I preferred and understand better. It made me happy that Shona was available for me to understand deeply (C14201—paper; female; 24 years).

Despite the control arm being paper-based PIS, participants described the approach as interactive because they tended to elect for the researcher to read it to them. This meant that both approaches allowed for a dialogue between the researcher and the participant in which participants could ask questions that provided more clarification, which may have supported comprehension across both arms.

I liked reading, especially reading with the researcher because it helped me to understand the information faster (C23121—paper; male; 19 years).

The video is a good method as it is inclusive since it can accommodate those who cannot read, they just listen to the audio. (C14211—video; female; 21 years).

#### Engagement

There were two features, which affected participants’ engagement in the informed consent processes. The volume of information presented was a negative feature identified by participants in both arms. The vibrancy and novelty of the video were positively received by participants in the intervention arm and such advantages were not noted by those who used the paper-based PIS.

The volume of information was generally considered to be too burdensome to be manageably absorbed. This was common to both arms and undermined participants’ engagement with the study information. Neither format could overcome participants’ lack of enthusiasm for the volume of information being presented.

The information made a lot of sense though it took time to go through the paper (C21132—paper; female; 23 years).

Further, the video should be shortened because it is a bit too long which makes it difficult for one to continue paying attention. It should be at least 5 minutes instead of the 9 minutes that I saw (C14281—video; female; 19 years).

In addition to the high volume of the information presented in both formats, engagement was hampered by the unfamiliar words and cumbersome linguistic phrases, which needed to be used in the Shona and Ndebele languages to explain technical concepts and convey the appropriate information.

What I find difficult is some of the Shona terms which I didn’t understand like ’Njodzi Nekusagadzikana’ to me this is deep Shona terms (C13251—paper; male; 24 years).

*Njodzi Nekusagadzikana* translates to risks and discomfort in English. Another participant commented.

I had to ask for further clarification. The words written on that form are just deep, this is not the simple Ndebele that we read on WhatsApp (C21141—paper; male; 21 years).

The dynamism and imagery of the video were described as engaging and relatively easy to pay attention to. Participants highlighted that the research process and activities were easily imagined and grasped through watching the video. Participants in the intervention arm described how the use of visual imagery and music, as well as the pace and colour, enhanced young people’s attentiveness to engaging with the study information presented.

#### Trust and realism

The vibrancy of the videos also had the secondary effect of generating trust in the research team and study. This operated in two ways. First, the act of developing research material in a format to align with what young people might want and respond to (video-based) was interpreted as a demonstration that the study would be youth-centred and the research team would consider and be respectful of their needs throughout the research.

I think a lot of youth would like video because videos trend, that’s what draws most of the youth attention. (C23261—video; male; 22 years).

After seeing the video, and various photos of CHIEDZA staff, I am certain that it is the study for the youth and I would like to participate. (C13241—video; female; 18 years).

Second, as the video showed the research venues and portrayed the actual research procedures, it conferred legitimacy onto the study. This illuminated an implicit concern that participants may assume that the information presented could be describing a hoax study. Watching the study procedures in the video in familiar venues or settings, meant they felt confident that it was genuine, which in turn enhanced their trust in the study and its approach.

The video shows what the CHIEDZA people have been doing, which built my trust and because of that I was able to see that this is a genuine study (C14231—video; female; 18 years).

There is proof that the study is real since I am watching the people telling me what I am expected to do in the video. I can also manage to trust the organization which is doing the study (C11791—video; female; 21 years).

The transparent portrayal of the research process in following a young person through the steps in the study, served to make the study ‘real’ to the participants. They described being able to base their decision to participate on their understanding of what it would ‘actually be like’ rather than having to imagine it. This made the information on which they were basing their decision to participate both more credible and more reliable. This included a heightened awareness of the risks involved in participation. For example, those watching the video appeared to focus more on the finger prick necessary for the blood sample collection, a process demonstrated in the video, which received comparatively less attention from those in the control arm. This caused some concern about the perceived pain and wound from the finger prick: *I didn’t like the fact that it is stated that there will be Dry Blood Sample collection, pricking is painful and can cause a wound. (C13221—video; female; 24 years)*. However, this served to enhance their awareness of this activity within the research, a key objective of the informed consent process.

#### Adaptability

Although the video had been designed specifically to appeal to young people, participants emphasised how valuable the video could potentially be in meeting the needs of other particular groups, whom they considered to be more in need of alternative, accessible formats through which to engage in research. They characterised the tool as being especially pertinent for use with key population with low literacy, such as the elderly, or people living with disabilities, for whom the video could be adapted with subtitles or sign language to be more inclusive of those with hearing impairments:

Those young people who cannot see can easily get to understand about the study as they can get all the information by just listening to the video. The video can also work on the elderly particularly those who cannot read (C14231—video; female; 18 years).

You can also add some subtitles and sign language to cater for the deaf (C21191—video; male; 19 years).

## Discussion

Our study demonstrates that comprehension of study information conveyed using video-based information is non-inferior to using the conventional approach that uses paper-based information sheets and consent. However, the qualitative findings indicated that there may be areas such as engagement, trust and its potential adaptability in which the video consent approach may be superior.

Overall, the total comprehension score obtained was relatively high. These high scores could have been a result of participants paying more attention to the study information as they were informed that their comprehension would be assessed as part of the trial procedures.

Education level and main activity status were not associated with comprehension in both arms. A possible explanation is that the researcher engaged with participants by taking more time to explain research information and answer questions in both the intervention and control arms such that those who had lower literacy managed to receive the clarity they required before the comprehension assessment. However, comprehension scores in the control arm may have been higher in comparison to comprehension that would be obtained in other research settings given that comprehension was being assessed as part of a trial, and research assistants may have made more effort to explain the procedures. Previous participation was associated with better comprehension in the control arm. This may be due to familiarity with the format of the traditional consent form, which may have resulted in better comprehension in this group.

On the other hand, intervention arm participants who were younger and lived in Harare demonstrated better comprehension compared with their counterparts. Harare is the capital city and research studies are likely to be more common than in Bulawayo (450 km west of Harare) or in Mashonaland East, which includes both peri-urban and urban settings. Better understanding in Harare may likely be due to possibly more familiarity with research and possibly better overall literacy. There was, however, no evidence of an association of comprehension with previous study participation among those in the video consent arm, which may indicate the novelty of the method in this study setting.

Both consent methods were highly acceptable in terms of being interactive and inclusive. Key to the apparent equivalence in both arms may have been that participants opted to have the PIS read to them by the researcher, facilitating a more conversational approach to the consenting process compared with relying on the eligible individual to read the PIS alone. However, to achieve this parity requires a consistent delivery of best practice through attentive research teams. The video method may provide greater consistency in the engaging presentation of information, which in turn may potentially support enhanced comprehension.

In our trial, both methods were supported by the active role of the researcher in answering questions prompted by watching the video or listening to the PIS be read to them. We consider this to be an essential component of a comprehensible and acceptable consenting process. This aligns with research by Anderson *et al,* which reports that although the use of interactive technology is likely to improve the informed consent process, it cannot replace the human connection that is central to the process.^17^ If the broader aim is to improve engagement in the consent process, there may be considerable potential for studies to be designed to give a choice between video and paper-based consent processes to eligible study participants.

In this study, the average time taken to complete the consent process using the video information was longer than using the paper-based method. Despite the relative advantages of the video method to convey the information in a more engaging way, across both arms, the volume of information presented was considered burdensome by participants.

Engagement was hampered by the use of complex Shona and Ndebele words. This may be due to the process of translating English information sheets to vernacular, which is usually difficult and can result in the loss of the original simplified text. Videos offer the opportunity to show research processes and thus complex terminology can be minimised, unlike in written information sheets. Flory *et al*^15^ and Mack *et al*^29^ argue that despite the recognition of the need to make consent documents shorter or more readable, they continue to increase in length and complexity. This illuminates a dilemma. If the information presented is so comprehensive that its extensiveness exceeds what individuals can reasonably absorb and digest, then we risk losing the attention and engagement of participants overall and participation decisions are made without appropriate comprehension and consideration.

The use of the video may not sufficiently address the broader challenge of low literacy. The requirement to include such extensive information may perversely further compromise the extent to which participants can make informed decisions to participate in research. Alongside use of videos as a more creative format to convey information, there is a need for ethical boards and researchers to consider how to reduce and refine the content and volume of information necessary for inclusion in the informed consent process so that it is context-appropriate and age-appropriate and consequently support genuine comprehension and informed consent. Bwakura-Dangarembizi *et al* reported the inaccessibility of research studies due to ethical and legal frameworks, which do not take into account the cultural context in Zimbabwe, warranting involvement of critical stakeholders such as communities representing the target population in the development of consent procedures.^30^

In addition to the video being non-inferior to the paper-based consent method, the video presented indirect advantages including evoking trust from the participants. Resnik explains that where informed consent and trust are concerned, the research participants often rely on investigators to help them understand important study information in the consent process.^1^ The video, in combination with the researchers’ presence to facilitate and answer questions, appears to be a particularly strong combination to support participants’ comprehension and trust in the research process. As indicated by the young people themselves, the video format has considerable potential for adaptation to tailor the delivery of content to meet the differentiated needs of particular groups such as people living with disabilities and the elderly.

The video information was coupled with obtaining consent on a tablet. An advantage of digital methods is reducing the amount of paper used and reduced requirements for physical data storage.

The study has some limitations. The comprehension questionnaire to ascertain the primary outcome was developed for this study and has not been validated or piloted. Furthermore, all questionnaire items were allocated the same score weighting, although some aspects of information may potentially be more important to understand than others. Comprehension assessed immediately after receiving information is likely to be high due to the immediate information recall, which may not reflect true comprehension. A research study by Ndebele *et al* investigating participants’ understanding of clinical trial concepts reported that most of the trial participants had good knowledge of the concepts but had inadequate understanding of what the concepts entailed suggesting that the consent obtained from these participants may have not been truly informed.^31^ Further investigation on study information comprehension would need to include later time intervals such as 7 or 14 days after receiving information for optimal validation of the questionnaire.

Current informed consent research studies are limited to the evaluation of comprehension of clinical trial study information and this limits the comparability of our findings. The trial design meant that none of the participants engaged in both formats. The qualitative discussions therefore could only focus on a participant’s experience of one method and there were no opportunities for participants themselves to make a comparison. The analysis presented is a comparison of the participants’ accounts. Finally, as study staff were not blinded to the trial, they may have taken more care to deliver the written information more carefully.

Given that our participants are youth, their reading rate may be faster than for older adults. However, there would be variations in the reading rate due to difference in literacy levels and speaking English as a second language.

### Conclusion

Comprehension of study information using the video-based information method is non-inferior to the paper-based consent method. While both methods were acceptable to participants, the video-based method had advantages in improving engagement and building trust. These factors, alongside its inherent adaptability, demonstrate its potential value in supporting the informed consent process for specific population groups, in particular those with low literacy, people living with disabilities and youth. The video consent method shows considerable promise to be used as part of a person-centred, context-sensitive approach to enhance the informed consent process across a broad range of population groups and should be considered as a valuable option for research teams and institutional review boards.

## supplementary material

10.1136/bmjgh-2023-014481 online supplemental file 1

10.1136/bmjgh-2023-014481 online supplemental file 2

10.1136/bmjgh-2023-014481 online supplemental file 3

10.1136/bmjgh-2023-014481 online supplemental file 4

10.1136/bmjgh-2023-014481 online supplemental file 5

## Data Availability

Data are available upon reasonable request.
