# Supplementary figures and images for: Acceptability and effectiveness of a study information video in improving the research consent process for youth: a non-inferiority trial
Source: BMJ Glob Health. 2025 Jan 11;10(1):e014481. doi: 10.1136/bmjgh-2023-014481 (PMC11749567; doi:10.1136/bmjgh-2023-014481)

**Supplementary Figure 1: Consent Procedures Trial Study Design**

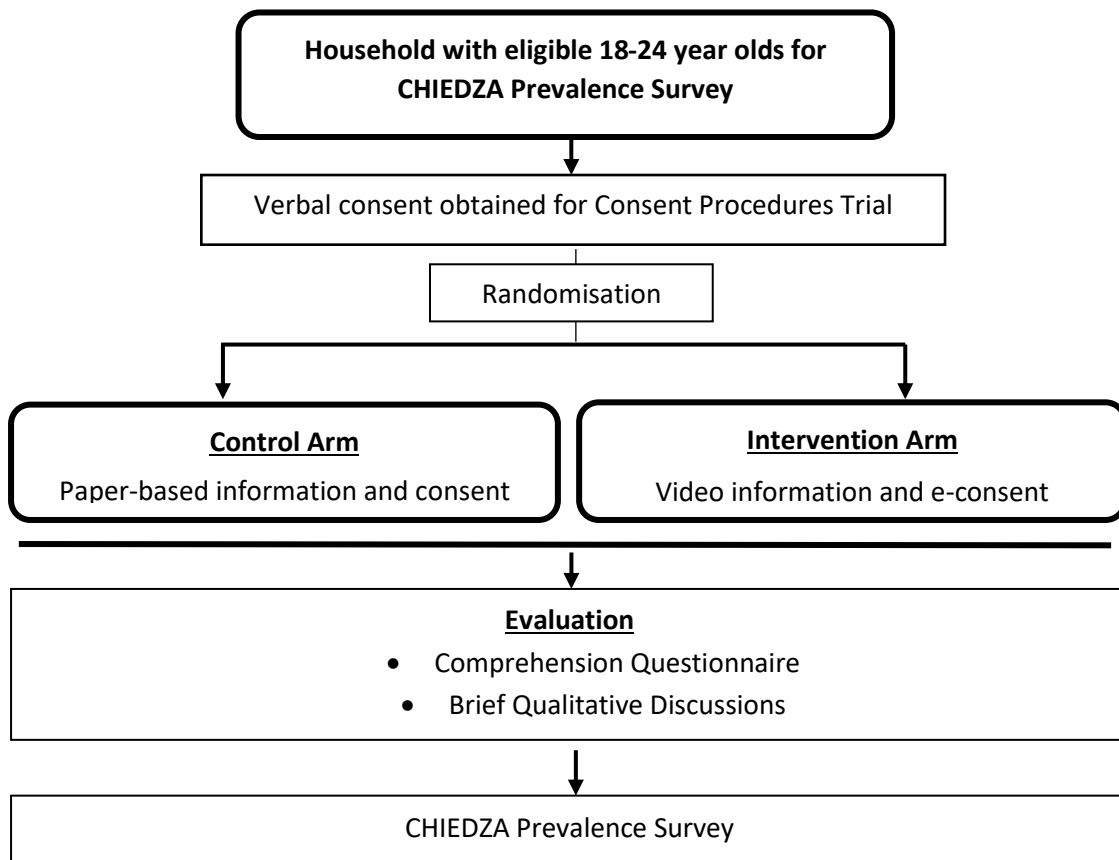

Supplement: online supplemental file 1 [file bmjgh-10-1-s001.pdf]

Supplementary Figure 2: Time taken to complete consent process by study arm

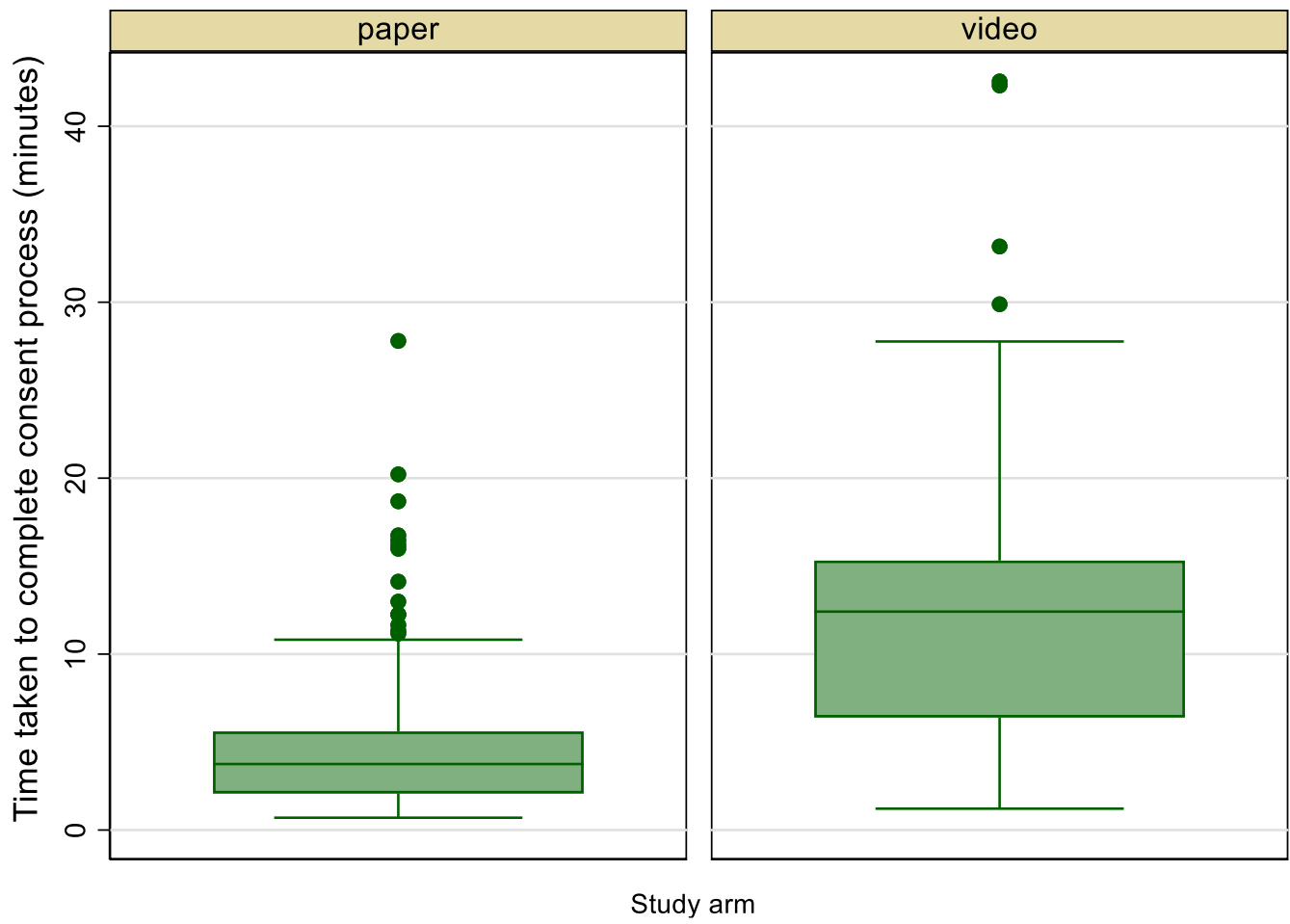

Supplement: online supplemental file 2 [file bmjgh-10-1-s002.pdf]
