# Supplementary material for: Acceptability and effectiveness of a study information video in improving the research consent process for youth: a non-inferiority trial
Source: BMJ Glob Health. 2025 Jan 11;10(1):e014481. doi: 10.1136/bmjgh-2023-014481 (PMC11749567; doi:10.1136/bmjgh-2023-014481)
Supplement: online supplemental file 3 [file bmjgh-10-1-s003.pdf]

**Supplementary Table 1: Comparison by arm of those with correct comprehension scores of specific consent principles**

| Questionnaire items | Total (N=921 [100%]) | Intervention (N=494 [53.6%]) | Control (N=427 [46.4%]) |
|---|---|---|---|
| **Reason for participation invite** | 874 (94.9) | 406 (95.1) | 468 (94.7) |
| **Withdrawal without penalty** | 834 (90.6) | 395 (92.5) | 439 (88.9) |
| **Reason for signing study consent form** | 786 (85.3) | 369 (86.4) | 417 (84.4) |
| **Monetary reimbursement for participation** | 833 (90.5) | 384 (89.9) | 449 (90.9) |
| **Whether participation is by choice** | 834 (90.5) | 392 (91.8) | 442 (89.5) |
| **Provision of study team contact** | 842 (91.4) | 384 (89.9) | 458 (92.7) |
