# Supplementary material for: Acceptability and effectiveness of a study information video in improving the research consent process for youth: a non-inferiority trial
Source: BMJ Glob Health. 2025 Jan 11;10(1):e014481. doi: 10.1136/bmjgh-2023-014481 (PMC11749567; doi:10.1136/bmjgh-2023-014481)
Supplement: online supplemental file 4 [file bmjgh-10-1-s004.pdf]

Supplementary Table 2: Comparison of comprehension survey and response options by arm

Question	Response option	Control N=427 (%)	Intervention N=494 (%)
**1. Which of the following statements is true?**			
1.1 You have been invited to take part in the CHIEDZA study because:	You did not participate in any of the CHIEDZA services	4 (0.9)	4 (0.8)
	You are aged between 18 and 24 years	406 (95.3)	468 (94.7)
	You participated in the CHIEDZA services	13 (3.1)	10 (2.0)
	You are aged above 30 years	0	0
	Can't remember	3 (0.7)	12 (2.4)
1.2 If you give written consent to take part in the study and later change your mind	You can withdraw within 3 months of giving written consent, but not after	1 (0.2)	8 (1.6)
	You can withdraw anytime without penalty	395 (92.5)	439 (89.6)
	You can withdraw anytime but will have to pay a penalty	5 (1.2)	7 (1.4)
	You cannot withdraw once you have given a written consent	12 (2.8)	19 (3.9)
	Can't remember	14 (3.3)	17 (3.5)
**2. Which of the following procedures will be done if you take part in the CHIEDZA study?**			
2.1 Giving a fingerprint	No	14 (3.3)	15 (3.1)
	Yes	403 (94.6)	469 (95.3)
	Don't know	9 (2.1)	8 (1.6)
2.2 Giving a sputum sample	No	325 (85.8)	395 (88.7)
	Yes	31 (8.2)	36 (8.1)
	Don't know	23 (6.1)	14 (3.2)
2.3 Measurement of height	No	29 (6.9)	38 (7.9)
	Yes	384 (91.4)	439 (91.5)
	Don't know	7 (1.7)	3 (0.6)
2.4 Answering questions about your health	No	67 (16.5)	96 (20.3)
	Yes	334 (82.3)	375 (79.3)
	Don't know	5 (1.2)	2 (0.4)
2.5 Measurement of blood pressure	No	36 (8.8)	32 (6.6)
	Yes	366 (89.7)	447 (92.2)
	Don't know	6 (1.5)	6 (1.2)
2.6 Measurement of oxygen levels	No	349 (91.4)	412 (91.2)
	Yes	20 (5.2)	29 (6.4)
	Don't know	13 (3.4)	11 (2.4)
2.7 Taking an X-Ray	No	357 (94.2)	421 (95.5)
	Yes	12 (3.2)	14 (3.2)
	Don't know	10 (2.6)	6 (1.3)
2.8 Measurement of weight	No	20 (4.8)	21 (4.3)
	Yes	390 (93.8)	462 (95.3)
	Don't know	6 (1.4)	2 (0.4)
2.9 Blood sample collection	No	12 (2.8)	22 (4.5)
	Yes	410 (97.2)	468 (94.9)
	Don't know	0	3 (0.6)
**3. What will be done with the blood sample that is collected?**			
3.1 HIV test	No	8 (1.9)	9 (1.8)
	Yes	418 (98.1)	481 (97.8)
	Don't know	0	2 (0.4)
3.2 Hepatitis B test	No	363 (94.0)	428 (93.4)
	Yes	11 (2.9)	15 (3.3)
	Don't know	12 (3.1)	15 (3.3)
3.3 HIV viral load test	No	110 (26.8)	130 (27.0)

	Yes	292 (71.0)	337 (69.9)
	Don't know	9 (2.2)	15 (3.1)
3.4 Blood sugar test	No	357 (91.5)	412 (91.0)
	Yes	22 (5.6)	31 (6.8)
	Don't know	11 (2.8)	10 (2.2)
3.5 Liver function test	No	375 (96.2)	435 (95.4)
	Yes	8 (2.1))	16 (3.5)
	Don't know	7 (1.7)	5 (1.1)
3.6 Herpes test	No	359 (92.1)	433 (95.0)
	Yes	19 (4.9)	15 (3.3)
	Don't know	12 (3.0)	8 (1.7)
3.7 Storage in a lab for future research studies	No	84 (20.7)	100 (21.1)
	Yes	315 (77.6)	361 (76.3)
	Don't know	7 (1.7)	12 (2.6)
**4. If you want to know your HIV status, you**			
	Can contact the research team a week after your interview to come and do a test	6 (1.4)	11 (2.2)
	Go to the nearest clinic to get an HIV test free of charge	397 (93.4)	441 (89.3)
	Contact the research team who will give you the result of the blood test they take	11 (2.6)	20 (4.1)
	Contact the research team 3 months after your interview to come and do a test	3 (0.7)	8 (1.6)
	Can't remember	8 (1.9)	14 (2.8)
**5. Why will you be asked to give us your fingerprint on a scanner?**			
	To show that you have agreed to take part in this study	32 (7.5)	45 (9.1)
	To cross-check whether you ever attended any CHIEDZA services	363 (85.2)	394 (79.8)
	To identify you to others	5 (1.2)	13 (2.6)
	To record your age	1 (0.2)	0
	Can't remember	25 (5.9)	42 (8.5)
**6. Who will have access to your health information?**			
6.1 Members of the CHIEDZA research team	No	45 (10.6)	54 (11.1)
	Yes	376 (88.5)	429 (87.9)
	Don't know	4 (0.9)	5 (1.0)
6.2 My family members	No	376 (97.2)	442 (97.1)
	Yes	3 (0.8)	9 (2.0)
	Don't know	8 (2.0)	4 (0.9)
6.3 Anyone working at the Biomedical Research and Training Institute	No	222 (55.6)	264 (57.3)
	Yes	161 (40.4)	176 (38.2)
	Don't know	16 (4.0)	21 (4.5)
6.4 External researchers who do not work at Biomedical Research and Training Institute but who have approval from the Medical Research Council of Zimbabwe	No	155 (37.8)	176 (37.3)
	Yes	238 (58.1)	282 (59.8)
	Don't know	17 (4.1)	14 (2.9)
6.5 My Employer or School (if I am in school)	No	382 (96.7)	441 (96.5)
	Yes	7 (1.8)	10 (2.2)
	Don't know	6 (1.5)	6 (1.3)
**7. What does it mean when you sign the study consent form?**			
	You would like to take part in similar studies	19 (4.5)	36 (7.3)
	You do not want to take part in this study	34 (8.0)	34 (6.9)

	You are agreeing to take part in this study	369 (86.4)	417 (84.6)
	None of the above	2 (0.5)	4 (0.8)
	Can't remember	3 (0.7)	2 (0.4)
**8. Will you receive money for taking part in the study?**			
	No	384 (89.9)	449 (91.1)
	Yes	40 (9.4)	38 (7.7)
	Don't know	3 (0.7)	6 (1.2)
**9. Is participation in this study by choice?**			
	No	35 (8.2)	50 (10.2)
	Yes	392 (91.8)	442 (89.8)
	Don't know	0	0
**10. Can you still take part in the survey but refuse to get your blood samples stored?**			
	No	38 (8.9)	57 (11.6)
	Yes	370 (86.7)	422 (86.0)
	Don't know	19 (4.4)	12 (2.4)
**11. Will you be given a phone number of someone to contact if you have any questions about the study?**			
	No	32 (7.5)	31 (6.3)
	Yes	384 (90.1)	458 (92.9)
	Don't know	10 (2.4)	4 (0.8)

Correct responses
