# Supplementary material for: Acceptability and effectiveness of a study information video in improving the research consent process for youth: a non-inferiority trial
Source: BMJ Glob Health. 2025 Jan 11;10(1):e014481. doi: 10.1136/bmjgh-2023-014481 (PMC11749567; doi:10.1136/bmjgh-2023-014481)
Supplement: online supplemental file 5 [file bmjgh-10-1-s005.pdf]

Supplementary Table 3: Distribution of baseline characteristics of the qualitative discussion participants by study arm

Characteristic	Intervention (N=47)	Control (N=43)
**Age (years)**		
**18-20**	29 (61.7)	17 (39.5)
**21-24**	18 (38.3)	26 (60.5)
**Sex**		
**Female**	32 (68.1)	23 (53.5)
**Male**	15 (31.9)	20 (46.5)
**Province**		
**Harare**	32 (68.1)	28 (65.1)
**Bulawayo**	10 (21.3)	10 (23.3)
**M. East**	5 (10.6)	5 (11.6)
**Highest education level**		
**Primary**	2 (4.30)	6 (13.9)
**Secondary**	44 (93.6)	34 (79.1)
**Tertiary**	1 (2.10)	3 (7.00)
**Main activity**		
**None**	26 (55.3)	25 (58.1)
**Going to school**	10 (21.3)	11 (25.6)
**Employed**	3 (6.40)	2 (4.70)
**Informal work**	8 (17.1)	5 (11.6)
**Previous research study participation**		
**No**	31 (66.0)	24 (55.8)
**Yes**	16 (34.0)	19 (44.2)
